# Molecular epidemiology of genogroup II norovirus infections in acute gastroenteritis patients during 2014–2016 in Pudong New Area, Shanghai, China

**DOI:** 10.1186/s13099-018-0233-1

**Published:** 2018-02-23

**Authors:** Caoyi Xue, Lifeng Pan, Weiping Zhu, Yuanping Wang, Huiqin Fu, Chang Cui, Lan Lu, Sun Qiao, Biao Xu

**Affiliations:** 1 School of Public Health, Fudan University, Shanghai, 200032 China; 2 Shanghai Pudong New Area Center for Disease Control and Prevention, 3039 Zhangyang Road, Shanghai, 200136 China; 3 Fudan University Pudong Institute of Preventive Medicine, Shanghai, 200136 China

## Abstract

**Background:**

Norovirus (NoV), a member of the Caliciviridae, is now recognized as the leading cause of acute gastroenteritis (AGE) worldwide. Globally, the GII.4 Sydney_2012 variant has predominated in NoV-related AGE since 2012, although the novel variant GII.17 has also been reported as responsible for gastroenteritis outbreaks in East Asia since 2014. This study aimed to disclose the recent genotype patterns of NoV genogroup II (GII) presenting in AGE patients in Pudong New Area of Shanghai through a laboratory-based syndromic surveillance system. The study further aimed to delineate the predominant strains circulating in the population.

**Methods:**

Pudong New Area is located in eastern Shanghai and covers 20.89% of the Shanghai population. The laboratory-based syndromic surveillance system is composed of 12 sentinel hospitals among the 68 general hospitals in this area. AGE patients who sought medical care were sampled following an AGE surveillance protocol. Stool samples were collected from participating patients, and a standardized questionnaire was given to each patient by trained nurses to gain information on the disease profiles and demographics of the patients. Real-time reverse transcription polymerase chain reaction (qRT-PCR) was used to screen the GI nd GII NoV and RT-PCR was used to amplify NoV GII partial capsid protein open reading frame 2 (ORF2). NoV Genotyping Tool (version 1.0, RIVM, MA Bilthoven, Netherlands) was used for genotyping, and a phylogenetic analysis was conducted by MEGA 7.0.

**Results:**

During 2014–2016, among the 2069 virus-infected AGE cases, 65.88% were caused by NoV. NoV-AGE occurred most frequently in the periods from October to March. The patients with more severe diarrheal symptoms and vomiting were more likely to be infected by NoV. The main genotypes were GII.17 (44.69%) and GII.4 (39.26%), which dominated the NoV-AGE epidemics jointly or in turn, whereas a slight increase in GII.2 was observed beginning in May 2016. The GII.17 strains tended to cluster more with the Hu/JP/2014/GII.P17_GII.17/Kawasaki323 variants, representing novel prevalent strains. Among the GII.4 strains, the GII.4 Sydney_2012 variant was still the predominant strain.

**Conclusions:**

NoV GII has become the main cause of virus-infected AGE in Pudong New Area, Shanghai. The predominant genotypes of NoV GII were GII.17 and GII.4. Comprehensive laboratory-based surveillance is important for clinical diagnosis and treatment. Identification of emerging new genotypes is also crucial for the prevention and control of NoV-infected AGE.

## Background

Acute gastroenteritis (AGE) is a major cause of morbidity and mortality affecting both developing and developed countries [1, 2]. AGE is ranked second among all infectious diseases, causing an estimated 89.5 million disability adjusted life years (DALYs) and 1.45 million deaths worldwide per year [2].

Pudong New Area is located in eastern Shanghai with the population of 5.5 million, covering 20.89% of the Shanghai. To support early detection of and response to AGE epidemics, a laboratory-based syndromic surveillance system was established in 2010 in Pudong New Area of Shanghai. Based on service load, laboratory capacity, and geographic location, 12 of the 68 general hospitals in this area were selected as sentinel hospitals for AGE syndromic surveillance. The surveillance system includes three tertiary general hospitals, seven secondary general hospitals and three community health care centers covering about 71.2% AGE patients per year.

Norovirus (NoV), a member of Caliciviridae, is now recognized as the leading cause of AGE worldwide [3]. The first report of a NoV-AGE outbreak was in 1968 in the United States. Since then, NoV-AGE has become epidemic both in developed and developing countries, especially among children and the elderly. In Shanghai, according to the surveillance data, outbreaks occurred in schools and workplaces every year [4, 5]. These outbreaks could involve tens or even hundreds of patients. Thus, understanding the epidemiology of NoV-AGE and enhancing pathogen-guided intervention is of great importance.

Norovirus is currently classified into five genogroups, among which only NoV GI, G GII and GIV are associated with human gastroenteritis [6]. The genome contains 3 open reading frames (ORFs). ORF1 encodes non-structural proteins that include an RNA-dependent RNA polymerase (RdRp); ORF2 encodes a capsid protein (VP1); and ORF3 encodes a minor capsid protein (VP2). NoV G GII is known to have a wider circulation than that of NoV GI, playing a major role in AGE. Based on differences in capsid protein VP1, NoV G GII can be further classified into 23 genotypes, namely, GII.1–GII.23 [7].

Norovirus circulate widely and hold considerable genetic diversity. NoV GII.4 has become the most common genotype circulating globally [8] and represents a typical example of NoV diversity. However, since the autumn of 2014, a new GII.17 strain has emerged as the major cause of AGE outbreaks. The emergence of new NoV genotypes suggests further threats of outbreaks all over the world. Moreover, some studies have found that the newly emerging strains might have stronger invasion abilities and target people with impaired immunity through displacing the previous predominant genotype [9].

The average annual incidence for NoV-associated outpatient visits was estimated to be 1.5/100 person-years with diarrhea in the community who sought medical care and a community incidence of 8.9/100 person-years by conducting the age-stratified Hospital Utilization Attitudes Survey among respondents residing in Pudong. NoV was a substantial burden on the community and healthcare system of Pudong [10]. Thus, understanding the dominant genotypes of NoVs and identifying longitudinal variation in circulating NoVs are key to NoV-AGE diagnosis, vaccine development, and outbreak prevention and control. This study aimed to study the molecular epidemiology of GII NoV presenting in AGE patients in Pudong New Area of Shanghai through a laboratory-based syndromic surveillance system and, furthermore, to delineate the dominant strains circulating in the population in recent years. Findings from this study could be useful in the early detection of and rapid response to potential NoV-AGE outbreaks.

## Methods

### Study participants and specimen collection

From January 1, 2014, to December 12, 2016, in each sentinel hospital every week, the first two to five AGE patients who sought medical care at the diarrhea outpatient department were identified for pathogenic-based surveillance. According to the definition given by the World Health Organization [11] and Diarrheal Syndrome Surveillance Protocol [12], the requirements for AGE reporting are those visiting diarrhea outpatient department with 3 or more loose or liquid stools per day. Each participant should have a 2-week symptom-free period before the episode. The AGE cases of suspected or confirmed outbreaks were excluded. Each patient was requested to submit one stool sample for bacterial culture and viral detection following the routine diagnosis procedure of AGE. The specimens were sent to the Pudong New Area Center for Disease Control and Prevention (PDCDC) within 24 h.

### RNA extraction

Nucleic acids were extracted from a 200 μL suspension using a QIAxtractor TM workstation (Qiagen, Hilden, Germany) with QIAxtractor Virus Reagents Qiagen, Maryland, USA) according to the manufacturer’s instructions. The elution volume used for the nucleic acids was 50 μL.

### Primers and probes

Real-time reverse transcription polymerase chain reaction (qRT-PCR) was used for initial sample testing for NoV according to the method of previous literature [13]. Genes for sequencing were amplified by RT-PCR from those qRT-PCR positive samples. The primer pairs and probes used for sample screening and sequencing of NoV are listed in Table 1.

**Table 1:** Primers and probes used to detect and sequence NoV GII

	Primer/probe sequence^a,b^		Location of 5′ end	References
qRT-PCR	F	CARGARBCNATGTTYAGR TGGATGAG	5003	[13]
	R	TCGACGCCATCTTCATTCACA	5100	
	P	TGGGAGGGCGATCGCAATCT	5048	
RT-PCR	F	CNTGGGAGGGCGATCGCAA	5058	[14]
	R	CCRCCNGCATRHCCRTTRTACAT	5401	

^a^Mixed bases are as follows: Y, C or T; R, A or G; B, not A; N, any

^b^F, R and P represent forward primer, reverse primer and oligonucleotide capture probe, respectively

### Screeing for GI and GII NoV

NoV GI and GII positive samples were screened with QuantiTect^®^ Probe RT-PCR Kit (Qiagen, Hilden, Germany). Each 20 μL reaction volume consisted of 5.7 μL RNase-free water, 12.5 μL 2× QuantiTect Probe RT-PCR Master Mix, 0.3 μL 10 mM TaqMan prober, 0.3 μL RT mix, 0.6 μL–10 mM e forward and reverse primers, respectively. qPCR was performed with a LightCycler^®^ 480II system (Roche, Switzerland) with the thermal profile of 50 °C for 30 min; 95 °C for 15 min, 40 cycles of 95 °C for 10 s, 56 °C for 1 min.

### Genotyping of GII NoV

The partial capsid protein open reading frame 2 (ORF2) of NoV GII positive sample was amplified by RT-PCR according to the method of previous literature [14]. RT-PCR was performed with an ABI9700 GeneAmp PCR system (ABI, Marsiling, Singapore) The reaction were carried out in a 20 μL reaction tube containing 5 μL 5× Qiagen OneStep RT-PCR Buffer, 1 μL Qiagen Onestep RT-PCR Enzyme Mix (Qiagen, Hilden, Germany) 12 μL of RNase-free water, 1 μL Dntp Mix, 0.5 μL–10 Mm forward and reverse primers, respectively. The thermal profile consisted of 50 °C for 30 min; 95 °C for 15 min, 40 cycles of 95 °C for 10 s, 52 °C for 1 min, 72 °C for 1 min; 72 °C for 10 min. PCR products were sized using QIAxcel DNA Screening Gel Cartridges (Qiagen, Hilden, Germany) on the QIAxcel system (Qiagen, Zurich, Switzerland), The expected PCR product size was 343 bp.

The amplicons were purified and sequenced by Shanghai Yiyue Biotechnology Co., Ltd. NoV genotypes were determined with NoV Genotyping Tool Version 1.0 (NGTV1, http://www.rivm.nl/mpf/norovirus/typingtool,RIVM,MA Bilthoven, Netherlands). Sequences of the strains that were isolated in the study were deposited in the GenBank database. The accession numbers are listed in Table 3 according to their genotypes based on the capsed region of ORF2. Alignment of the sequences was performed using BioEdit Sequence Alignment Editor software v7.0.9.0 [15]. The dendrogram was graphed by using the neighbour-joining method in MEGA version 7 [16]. Bootstrap resampling (1000 replications) was used, and bootstrap values ≥ 50% are shown.

### Statistical analysis

The Statistical Product and Service Solutions (SPSS v19.0) software (IBM, USA) was used for all analyses. Differences in discrete variable levels were examined using the Chi square test or the Fisher’s exact with two-tailed. A value of P < 0.05 was considered statistically significant.

## Results

### Detection trends of NoV infection in AGE patients

**Fig. 1:**
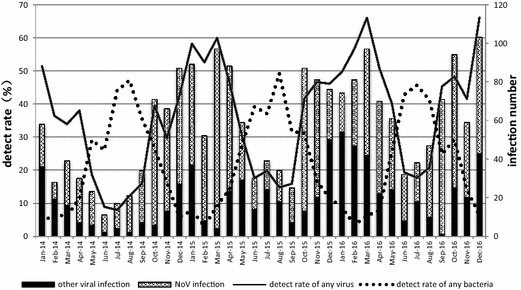
Detection rates of pathogens in AGE patients during 2014–16, Pudong New Area, Shanghai

In total, 5927 AGE patients were reported to the surveillance system during 2014–2016. The total detection rate of any intestinal virus was 34.91%, while 1363 NoV-AGE was detected, accounting for 65.88% of viral AGE cases. NoV-AGE occurred most frequently in the periods from October to March (Fig. 1). Among the AGE cases caused by NoV, the main genogroup was GII throughout these 3 years, while GI infection and double infection were rare (Table 2).

**Table 2:** Genogroups of NoV detected in AGE patients from 2014 to 2016 in Pudong New Area, Shanghai

Date	AGE cases	AGE NoV infections	GI		GII		Double infection with GI and GII	
	n		n	%	n	%	n	%
14-Jan	113	22	3	2.65	18	15.93	1	0.01
14-Feb	77	9	1	1.30	6	7.79	2	0.03
14-Mar	115	23	3	2.61	19	16.52	1	0.01
14-Apr	79	23	9	11.39	13	16.46	1	0.01
14-May	124	17	3	2.42	14	11.29	0	0.00
14-Jun	123	9	1	0.81	7	5.69	1	0.01
14-Jul	214	13	1	0.47	12	5.61	0	0.00
14-Aug	172	19	1	0.58	18	10.47	0	0.00
14-Sep	214	27	1	0.47	26	12.15	0	0.00
14-Oct	180	65	1	0.56	64	35.56	0	0.00
14-Nov	222	53	0	0.00	53	23.87	0	0.00
14-Dec	205	60	0	0.00	60	29.27	0	0.00
15-Jan	153	52	0	0.00	52	33.99	0	0.00
15-Feb	99	44	2	2.02	42	42.42	0	0.00
15-Mar	162	93	6	3.70	86	53.09	1	0.01
15-Apr	190	65	1	0.53	62	32.63	2	0.01
15-May	195	30	2	1.03	28	14.36	0	0.00
15-Jun	171	16	1	0.58	14	8.19	1	0.01
15-Jul	197	15	2	1.02	13	6.60	0	0.00
15-Aug	230	16	0	0.00	16	6.96	0	0.00
15-Sep	158	18	0	0.00	18	11.39	0	0.00
15-Oct	209	74	2	0.96	72	34.45	0	0.00
15-Nov	174	61	7	4.02	52	29.89	2	0.01
15-Dec	165	26	4	2.42	21	12.73	1	0.01
16-Jan	149	20	2	1.34	18	12.08	0	0.00
16-Feb	143	34	4	2.80	30	20.98	0	0.00
16-Mar	147	55	8	5.44	47	31.97	0	0.00
16-Apr	138	48	4	2.90	44	31.88	0	0.00
16-May	152	37	1	0.66	36	23.68	0	0.00
16-Jun	166	24	3	1.81	21	12.65	0	0.00
16-Jul	215	20	0	0.00	20	9.30	0	0.00
16-Aug	226	37	3	1.33	34	15.04	0	0.00
16-Sep	157	70	0	0.00	70	44.59	0	0.00
16-Oct	195	69	1	0.51	68	34.87	0	0.00
16-Nov	142	39	4	2.82	35	24.65	0	0.00
16-Dec	156	60	6	3.85	54	34.62	0	0.00

### Composition of GII NoV infection in AGE patients

**Fig. 2:**
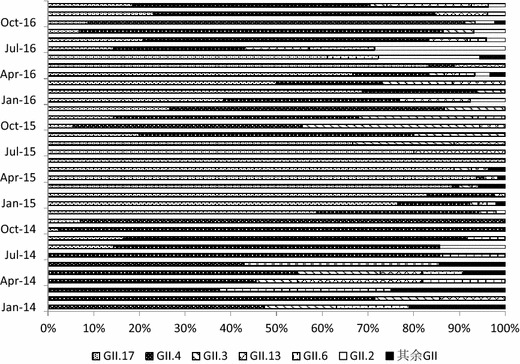
The composition of genogroup II NoV infection in the AGE patients (2014–2016)

A total of 866 NoV GII strains among 1363 GII NoV-AGE were further genotyped by sequencing the partial nucleotide sequence of ORF2. Twelve genotypes of NoV GII were determined by NoV Genotyping Tool Version 1.0 (Fig. 2). During the years from 2014–2016, the main genotypes were GII.17 (44.69%, 387/866) and GII.4 (39.26%, 340/866). These strains dominated the NoV-AGE epidemics jointly or in turn. The GII.17 strain was first detected in a case reported in Aug 2014, and it had replaced GII.4 as the dominant NoV genotype by Aug 2015. After that time, GII.4 and GII.17 predominated in turns from Sep 2015 to Dec 2016. Since June 2016, GII.2 has become more prevalent (Fig. 2).

### Epidemiological, clinical characteristics of NoV infection in the AGE patients

In the study, AGE cases were divided into five age groups, where 922 (52.6%), 530 (8.9%), 451 (7.6%), 3282 (55.4%), and 742 (12.5%) AGE patients fell into the age ranges of 0–5, 5–14, 15–64 and over 65 years, respectively. Among all the age groups, the detection rate of adults (25–64 years old) was highest (χ^2^ = 41.93, P < 0.001). The patients with more severe diarrheal symptoms and vomiting were more likely to be infected by NoV norovirus. The detection rates of NoV varies between different severity of diarrhea (χ^2^ = 15.68, P < 0.001). The patients with vomiting seems more likely to be infected by NoV (χ^2^ = 148.33, P < 0.001 (Tables 3, 4).

**Table 3:** Demographic characteristics of the NoV-AGE patients

	AGE patients	NoV-AGE patients	Detection rate [%, (95% *CI*)]	χ^2^	P value
	N (%)	N (%)			
Gender					
Male	3084 (52.0)	743 (54.5)	24.1 (22.6–25.6)	4.36	0.037
Female	2843 (48.0)	620 (45.5)	21.8 (20.3–23.3)		
Age group					
< 5 year	922 (15.6)	189 (13.9)	20.5 (17.9–23.1)	41.93	< 0.001
5–14 year	530 (8.9)	78 (5.7)	14.7 (11.7–17.7)		
15–24 year	451 (7.6)	99 (7.3)	22.0 (18.1–25.8)		
25–64 year	3282 (55.4)	847 (62.1)	25.8 (24.3–27.3)		
≥ 65 year	742 (12.5)	150 (11.0)	20.2 (17.3–23.1)		
Total	5927	1363	22.3

**Table 4:** Clinical manifestations of the NoV-AGE patients

	AGE patients	NoV-AGE patients	Detection rate [%, (95% *CI*)]	χ^2^	P value
	N (%)	N (%)			
Severity of diarrhea					
3–5 times per day	3752 (63.3)	801 (58.8)	21.3 (20.0–22.7)	15.68	< 0.001
> 5 times per day	2175 (36.7)	562 (41.2)	25.8 (24.0–27.7)		
Vomiting					
Yes	1398 (23.6)	489 (35.9)	35.0 (32.5–37.5)	148.33	< 0.001
No	4529 (76.4)	874 (64.1)	19.3 (18.1–20.4)		
Fever ( > 38 °C)					
Yes	833 (14.1)	196 (14.4)	23.5 (20.6–26.4)	0.15	0.693
No	5094 (85.9)	1167 (85.6)	22.9 (21.8–24.1)		
Dehydration					
Yes	43 (0.7)	9 (0.6)	20.9 (8.3–33.6)	0.10	0.747
No	5884 (99.3)	1354 (99.4)	23.0 (21.9–24.1)		

The genotype seems to have a role in the severity of diarrhea among different groups. GII.4 infection may caused less severe diarrhea symptoms (χ^2^ = 23.40, P < 0.001). Slight seasonal trend was observed in NoV GII.4 had a peak in prevalence in the autumn while GII.17 in the spring (χ^2^ = 308.71, P < 0.001). The time distribution showed minor seasonal variations across 3 years, respectively (χ^2^ = 151.73, P < 0.001) (Tables 5, 6, 7).

**Table 5:** Demographic characteristics of GII NoV-AGE patients with different genogroups

	GII.4 (n (%))	GII.17 (n (%))	Other GII (n (%))	χ^2^	P value
Age group (year)					
< 5	82 (24.1)	20 (6.2)	21 (15.1)	151.73	< 0.001
5–14	18 (5.3)	14 (3.6)	8 (5.8)		
15–24	18 (5.3)	20 (5.2)	13 (9.4)		
25–64	181 (53.2)	296 (76.5)	43 (30.9)		
> 65	41 (12.1)	37 (9.5)	54 (38.8)		
Gender					
Male	188 (55.3)	208 (53.7)	77 (55.4)	0.22	0.898
Female	152 (44.7)	179 (46.3)	62 (44.6)		
Total	340	387	139

**Table 6:** Seasonal distribution of GII NoV-AGE patients with different genogroups

	GII.4 (n (%))	GII.17 (n (%))	Other GII (n (%))	χ^2^	P value
Year					
2014	148 (43.5)	35 (9.0)	36 (25.9)	259.95	<0.001
2015	46 (13.5)	267 (67.0)	43 (30.9)		
2016	146 (43.0)	85 (22.0)	60 (43.2)		
Season					
Spring (Mar–May)	27 (7.9)	204 (52.7)	47 (33.9)	308.71	< 0.001
Summer (Jun–Aug)	33 (9.7)	38 (9.8)	26 (18.7)		
Autumn (Sep–Nov)	204 (60.0)	22 (5.7)	33 (23.7)		
Winter (Dec–Feb)	76 (22.4)	123 (31.8)	33 (23.7)		
Total	340	387	139

**Table 7:** Clinical manifestation of GII NoV-AGE patients with different genogroups

	GII.4 (n (%))	GII.17 (n (%))	Other GII (n (%))	χ^2^	P value
Severity of diarrhea					
3–5 times per day	224 (65.9)	201 (51.9)	62 (44.6)	23.40	< 0.001
> 5 times per day	116 (34.1)	186 (48.1)	77 (55.4)		
Vomiting					
Yes	129 (37.9)	163 (42.1)	57 (41.0)	1.34	0.510
No	211 (62.1)	224 (57.9)	82 (59.0)		
Fever ( > 38 °C)					
Yes	48 (14.1)	55 (14.2)	19 (13.7)	0.025	0.987
No	292 (85.9)	332 (85.8)	120 (86.3)		
Dehydration					
Yes	1 (0.3)	7 (1.8)	1 (0.7)	3.798	0.129
No	339 (99.7)	380 (98.2)	138 (99.3)		
Total	340	387	139		

### Nucleotide sequence accession numbers

The partial genomic NoV ORF2 nucleotide sequences of the strains that were isolated in this study were deposited in the GenBank database. The accession numbers are listed in Table 8 according to their genotypes based on the capsid region of ORF2.

**Table 8:** NoV GII strains genotyped with the capsid region of ORF2 from NoV-AGE patients in Pudong, Shanghai in 2014–2016

Genotype	Accession number of strains in this study	Quantity of strains	Strains in phylogenetic tree
GII.1	KU672081	1	KU672081
GII.2	KU672082, KU672083, KU672087, KY797735, KY983635-49	19	KU672087, U672082, KU672083, KY983635, KY983636, KY983637, KY983641, KY983643, KY983644, KY983647, KY983649, KY797735
GII.3	KU672088-92, KU672103-109, KY797764, KY983651-79	42	KU672103, KU672106, KU672107, KU672108, KU672088, KU672089, KU672090, KU672092, KY983651, KY983653, KY983654, KY983661, KY983668, KY983670, KY983671, KY983675, KY983677, KY983679, KY797764
GII.4	KU532332, KU532354-455, KU532676, KU532705-66, KY797698-725, KY983683-832	340	KU532440, KU532363, KU532368, KU532373, KU532376, KU532384 KU532386, KU532705, KU532709, KU532734, KU532758, KU532439, KU532441, KU532448, KU532454, KU532455, KY983692, KY983695, KY983704, KY983719, KY983728, KY983733, KY983737, KY983742, KY983747, KY983748, KY983750, KY983765, KY983798, KY983689, KY797711
GII.5	KU672135, KY797775	2	KU672135, KY797775
GII.6	KU672136, KU672149-59, KY797777, KY983833-39	20	KU672149, KU672151, KU672153, KU672156, KU672157, KU672158, KU672159, KU672136, KY983833, KY983835, KY983836, KY983838, KY983839, KY797777
GII.8	KU672163, KU672164, KY838340	3	KU672163, KU672164, KY983840
GII.12	KU672167, KU672168	2	KU672167
GII.13	KU672193-200, KU672170-177, KY983842-57, KY797778-81	36	KU672193, KU672194, KU672197, KU672200, KU672170, KU672171, KU672172, KU672173, KU672177, KY983842, KY983843, KY983845, KY983847, KY983850, KY983851, KY983853, KY983854, KY983856, KY797778
GII.14	KU532778, KU532779, KY983858	3	KU532778, KU532779, KY983858
GII.17	KU516833-966, KU516970-93, KU672203-331, KY797681-2, KY983864-961	387	KU516882, KU516947, KU516961, KU516979, KU516980, KU516881, KU672221, KU516883, KU672240, KU516922, KU516942, KU672318, KY983864, KY983868, KY983874, KY983875, KY983876, KY983879, KY983882, KY983887, KY983896, KY983908, KY983927, KY983941, KY983951, KY983956, KY983958, KY983961, KY797685
GII.21	KU532767-76, KY983962	11	KU532767, KU532768, KU532772, KU532776, KY983962

### Phylogenetic relationships among GII strains of NoV

**Fig. 3:**
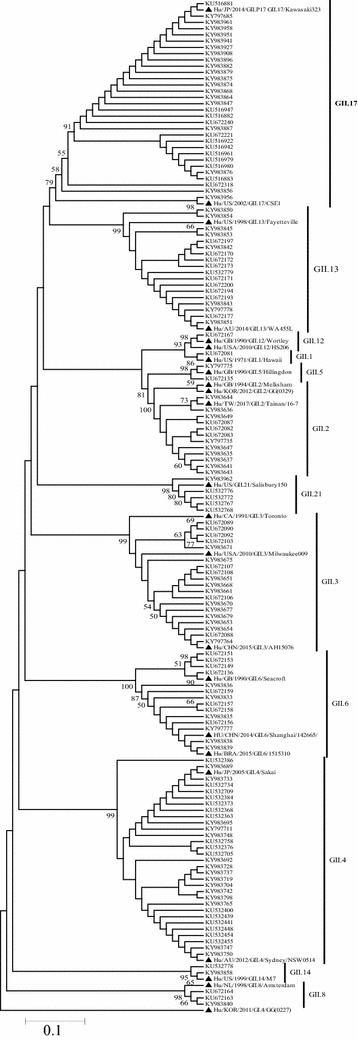
Phylogenetic tree based on partial capsid region (ORF2) of NoV strains. Sequences from sporadic acute gastroenteritis patients in Shanghai, 2014–2016 are shown with black triangles, time and Genbank accession numbers in the phylogenetic trees. Reference sequences, obtained from GenBank, display in the phylogenetic trees with black square, time, genotype, city or country, and accession number

A phylogenetic tree was constructed with 139 sequences from the strains collected and 24 additional worldwide reference sequences. The nucleotide sequences of the NoV ORF2 gene revealed that the GII.17 strains tended to cluster more with the strain Hu/JP/2014/GII.P17_GII.17/Kawasaki323 than with the strain Hu/US/2002/GII.17/CSE1, which suggested that the Kawasaki323 strain became the novel prevalent strain in Shanghai [17]. In the GII.4 group, the strain Hu/AU/2012/GII.4/Sydney/NSW0514 was more frequently observed than Hu/JP/2005/GII.4/Sakai was, indicating that the Sydney strain was still the predominant GII.4 strain in Shanghai (Fig. 3).

## Discussion

NoV infection is a leading cause of AGE among all age groups [18]. According to a systematic review of global NoV infection, the pooled prevalence of NoV infection in patients with AGE varied geographically, from approximately 19% (95% CI 16–22) in low-mortality developing countries to 20% (95% CI 17–22) in developed countries [19].

Given the high prevalence of NoV, representing a substantial disease burden to residents of all age groups, this study aimed to type the recent patterns of GII NoV presenting in AGE patients in Pudong through a laboratory-based syndromic surveillance system and to delineate the dominant strains circulating in the population. This 3-year data analysis and strain collection study could delineate the genotype circulation of NoV infection in AGE patients reported in Shanghai. The combination of epidemiological, etiological and molecular data analysis could provide comprehensive evidence to support NoV infection control.

In this study, the proportion of NoV in AGE patients was 21.59% in Pudong, close to that of developed countries. This result could be the result of a reduced number of bacterially-caused AGE cases, along with improvements in environmental and personal hygiene [20]. NoV-AGE was prevalent throughout the year and peaked in the cold season, as previously reported. In Pudong New Area, the seasonal increase of NoV-AGE started in October and usually lasted until March of the following year, consistent with the results of Xue et al. [21] in other districts in Shanghai and observations in other provinces in China [22, 23]. In the study, the adult aged from 25 to 64 years old seemed vulnerable to get NoV infection, which was not consistent with previous studies [19]. Since health seeking behaviour is voluntary related to the severity of the illness and accessibility of health service, most mild patients may appeal to Over-The-Counter Drug. On the other hand, vomiting is considered as the most frequent symptoms in NoV infected kids and infants. The frequency of vomiting without concurrent diarrhea suggests that epidemiology studies that enroll subjects based on the presence of diarrhea may be significantly underestimating the true burden of NoV infection, especially in the young people [24]. The patients with more severe diarrhea symptoms and vomiting were more likely to be infected by NoV. However, vomiting with concurrent diarrhea should be regarded as the predictors of NoV infection for more efficient and direct testing and treatment [25]. The circulating genotypes of NoV could vary geographically and longitudinally, which might influence the size and severity of NoV-AGE epidemics. Chen et al. [26] reported that, during the winter of 2014–2015, 6 GII genotypes circulated among adult patients in Shanghai, i.e., GII.2, GII.3, GII.4, GII.6, GII.13, and GII.17. Despite covering only a part of Shanghai, this study found 12 GII genotypes in Pudong New Area from 2014 to 2016. NoV GII.4 is the most common genotype globally, due to its high diversity, and a new strain of GII.4 emerges every 2–3 years, since it has a 1.7-fold higher rate of evolution on average within the capsid sequence and a greater number of non-synonymous changes compared to other NoVs [27]. A study by Lindesmith and colleagues found that the NoV GII.4 strains alter their histo-blood group antigen (HBGA) carbohydrate-binding targets over time, which allows for not only escape from high-penetrance host susceptibility alleles but also immune-driven selection in the receptor-binding region to facilitate avoidance of protective herd immunity [28]. This locus could represent a focal point for vaccine research to control NoV infection.

For example, the GII.4 strain US95/96 was responsible for a majority of the norovirus outbreaks in the US and Europe from 1995 to 1996. Between 2000 and 2004, US95/96 was replaced by two new GII.4 variants: Farmington Hills in the US and a new GII.4 variant, GII.4b, in Europe. In 2004, the Hunter GII.4 variant was detected in Australia, Europe, and Asia, and then this variant was replaced in early 2006 by two new cocirculating GII.4 variants, Sakai and Minerva, in the US, Europe, and Asia [28]. In 2009, the New Orleans strain was reported circulating worldwide [29].

In March 2012, a new GII.4 norovirus strain was identified in Australia and named GII.4 Sydney. As the predominant strain of the GII.4 genotype, Sydney_2012 and its variants have been widely reported since 2012 [30–32]. This emerging strain has caused AGE outbreaks in multiple countries [33]. GII.4 NoVs remain the predominant cause of outbreaks, and the GII.4 Sydney strain appears to have replaced the previously predominant strain, GII.4 New Orleans. Compared with other genotypes, GII.4 NoVs were associated with increased rates of hospitalization and death during outbreaks [34]. Similar to the results of Han et al., Sydney_2012, together with Den_Haag_2006b and New_Orleans_2009, were the main strains of GII.4 causing AGE in Shanghai, with Sydney_2012 and its variants being the predominant strains [35]. In this study, strain Hu/AU/2012/GII.4/Sydney/NSW0514 was more frequently observed than Hu/JP/2005/GII.4/Sakai was, indicating that the Sydney strain was still the predominant strain. Public health practitioners should remain alert to the potential for increased norovirus activity in future seasons related to variations in this strain. During the study period, we observed circulation of ORF2 genotypes that showed the trends in norovirus genomic diversity, recombination, and NoV reporting.

Since the winter of 2014, Jiangsu [36] and Zhejiang Province [35] in China have reported AGE outbreaks caused by a novel NoV genotype, GII.17. NoV GII.17 has a polymerase sequence and amino acid substitution in the capsid region, according to a study by Matesushiam et al. [37]. The novel GII.17 genotype was first detected in AGE patients in Aug 2014 in Pudong New Area [17], before the first report of an outbreak in Jiangsu. AGE outbreaks caused by the novel GII.17 genotype have also been reported from nurseries, schools and a military unit [38, 39]. NoV GII.17 could be detected in AGE patients, their close contacts and environmental surfaces such as trash cans and toilet bowls [5].

In this study, GII.2 infection was found to have increased slightly since June 2016. In late 2016, AGE outbreaks increased substantially in Pudong, greatly surpassing the numbers during the same season in previous years. Some studies reported that the majority of the outbreaks were associated with a new GII.2 strain that was likely to infect preschool children [40, 41]. From 2012 to 2016, a total of 313 NoV outbreaks in 24 provinces and 96 cities in China were reported to the National Emergent Public Health Event Information Management System; 109 (35%) were reported in 2016, mostly in winter. In November and December 2016, NoV outbreaks increased sharply, accounting for 56 (51%) of the 109 NoV outbreaks, and 78% of the GII.2 outbreaks occurred in kindergartens. Further analyses should be conducted to combine sporadic cases with outbreak data to explore the impact of the circulation of this new strain.

Although this study was surveillance-based, the findings of this study can only present the circulating and predominant NoV strains in the local area. Information on the epidemiological, clinical and molecular characteristics of the circulating and emerging strains was not linked. Additional efforts should be given to the study of NoV-AGE outbreaks and the transmission mechanism of this virus.

## Limitation

There were several limitations on our research. Firstly, the subjects were mainly selected from 12 sentinel hospitals selected through Modeling Spatial means of surfaces with stratified Non-homogeneity. The first two to five AGE patients who sought medical care at the diarrhea outpatient department were enrolled which would bring selection bias because of the convenient sampling. Secondly, the people under 14-year-old always visit the pediatric department while NoV usually cause vomiting among them, which would under-estimate the prevalence of the NoV infection among the under 14-year-old age group. Thirdly, genetic characteristics analysis of NoV was only based on partial polymerase regions (ORF2) in this study. Genotyping of RNA-dependent RNA polymerase regions should be conducted in order to enhance the accuracy and completeness of NoV GII genotyping which is our next research plan of the NoV infection surveillance. Meanwhile, the 3-year data of molecular epidemiological analysis of NoV infection has initiated in the year of 2014, which could only tracing out the circulating pattens rather than capturing the genomic diversity. This would also become our research focus in the future.

## Conclusions

NoV GII has become the main cause of virus-infected AGE in Pudong New Area, Shanghai. NoV-AGE accounted for 65.88% of viral AGE during 2014–2016, with GII as the main group; GII.17 and GII.4 dominated the NoV-AGE epidemics. GII.17 strains tended to cluster more with the strain Hu/JP/2014/GII.P17_GII.17/Kawasaki323, and GII.4 with Hu/AU/2012/GII.4/Sydney/NSW0514, as shown by the phylogenetic tree. A Comprehensive laboratory-based surveillance is important for clinical diagnosis and treatment. Identification of emerging new genotypes is also crucial for the prevention and control of NoV-infected AGE.

## Abbreviations

AGE: acute gastroenteritis
CI: confidence interval
NoV: norovirus
GI: genogroup I
GII: genogroup II
RT-PCR: reverse transcriptase polymerase chain reaction
